# Blood doping by cobalt. Should we measure cobalt in athletes?

**DOI:** 10.1186/1745-6673-1-18

**Published:** 2006-07-24

**Authors:** Giuseppe Lippi, Massimo Franchini, Gian Cesare Guidi

**Affiliations:** 1 Istituto di Chimica e Microscopia Clinica, Dipartimento di Scienze Morfologico-Biomediche, Università degli Studi di Verona, Verona, Italy; 2 Servizio di Immunoematologia e Trasfusione, Azienda Ospedaliera di Verona, Verona, Italy

## Abstract

**Background:**

Blood doping is commonplace in competitive athletes who seek to enhance their aerobic performances through illicit techniques.

**Presentation of the hypothesis:**

Cobalt, a naturally-occurring element with properties similar to those of iron and nickel, induces a marked and stable polycythemic response through a more efficient transcription of the erythropoietin gene.

**Testing the hypothesis:**

Although little information is available so far on cobalt metabolism, reference value ranges or supplementation in athletes, there is emerging evidence that cobalt is used as a supplement and increased serum concentrations are occasionally observed in athletes. Therefore, given the athlete's connatural inclination to experiment with innovative, unfair and potentially unhealthy doping techniques, cobalt administration might soon become the most suited complement or surrogate for erythropoiesis-stimulating substances. Nevertheless, cobalt administration is not free from unsafe consequences, which involve toxic effects on heart, liver, kidney, thyroid and cancer promotion.

**Implications of the hypothesis:**

Cobalt is easily purchasable, inexpensive and not currently comprehended within the World Anti-Doping Agency prohibited list. Moreover, available techniques for measuring whole blood, serum, plasma or urinary cobalt involve analytic approaches which are currently not practical for antidoping laboratories. Thus more research on cobalt metabolism in athletes is compelling, along with implementation of effective strategies to unmask this potentially deleterious doping practice

## Background

Ergogenic drugs are substances commonly used to enhance the athletic performance and include illicit drugs as well as a variety of compounds that are marketed as nutritional supplements. Although such drugs have been widely used by professional and elite athletes for centuries, research indicates that in recent years competitive athletes are increasingly experiencing with illicit compounds to improve both appearance and athletic abilities. Blood doping consists on techniques administered for non-medical reasons to healthy athletes to improve the blood oxygen carrying capacity, increasing oxygen deliver to the muscles, particularly in conditions of demanding physical exercise [1]. Owing to the favourable effects on endurance performance and recovery, blood doping has become rather popular among top class athletes over the past decades, as attested by numerous positive cases in antidoping controls [2]. Erythropoiesis-stimulating substances, namely recombinant human erythropoietin (rHuEpo), have been most used doping techniques since the mid 1980s [3]. However, the implementation of reliable laboratory tests to check for rHuEpo abuse using blood parameters and to identify rHuEpo and/or its metabolites in urine in 2001, might prompt some athletes to experiment with original and unpredictable doping strategies [3].

## Presentation of the hypothesis

Cobalt belongs to Group VIII of the periodic classification of elements and shares properties with nickel and iron. Cobalt is a naturally-occurring element with properties similar to those of iron and nickel. It has been reported that cobalt chloride promotes an hypoxia-like response, involving enhanced erythropoiesis and angiogenesis. The erythropoietin gene is the paradigm of oxygen-regulated genes controlled by the transcription factor Hypoxia Inducible Factor 1a (HIF-1a). HIF-1a is the key regulator of cellular and systemic oxygen homeostasis, through an increased DNA binding activity to the target erythropoietin gene sequence. Under normoxic conditions, the main mediator HIF-1α is rapidly degraded by the proteasome. During hypoxia or cobalt chloride administration, the degradation of HIF-1a is markedly inhibited. Therefore, HIF-1a binds to HIF-1b, crosses the nuclear membrane and powerfully activates the erythropoietin gene transcription [4].

Exposure to 120 or 150 mg/day of cobalt chloride results in the development of polycythemia with a substantial increase of hematocrit and hemoglobin, up to 20% above pre-treatment levels [5]. Thus, cobalt chloride might efficaciously prevent anemia in the clinical setting, inducing a marked and stable polycythemic response through a more efficient transcription of the erythropoietin gene, achievable at even moderate oral dosage of 30 mg/kg [4,6]. On this basis, we have recently hypothesized that cobalt chloride administration may be an alternative and dangerous blood doping technique, hardly detectable by current anti-doping testing policies [7]. Additionally, cobalt may also be suitable to enhance the erythropoietic response to low, maintenance doses of rHuEpo, which would not be detectable by current direct and indirect testing methods. Although little information is available so far on cobalt metabolism, reference value ranges or supplementation in athletes [8], there is emerging evidence that cobalt is used as a supplement and increased serum concentrations are occasionally observed in athletes [9], though there is no convincing proof for increased requirement or for any beneficial effect of specific supplementation on performance [10].

## Testing the hypothesis

Many athletes either are unaware of or do not consider the possible health risks caused by several doping techniques. Thus, cobalt misuse or abuse in athletes should be regarded in a critical perspective, along with gene doping targeted at enhancing expression and transcription of the erythropoietin gene [7,11]. Unnecessary cobalt salts administration produces adverse side effects, as cobalt accumulates in liver and kidney, promoting organ damage and dysfunction due to enhanced oxidative stress, even at the low dosage of 33.3 mg/kg [12]. Excessive cobalt in blood impairs thyroid activity and myocardial function, promoting carcinogenesis [12,13].

Owing to these severe and unpredictable side effects, doping by cobalt salts may reveal as a serious threat for the scientific community and for public health. Cobalt is easily purchasable, inexpensive and not currently comprehended within the World Anti-Doping Agency (WADA) prohibited list [14]. Unfortunately, routine cobalt testing within antidoping controls may result ineffective, due to its pharmacodynamic properties, the little data available on cobalt metabolism in athletes and the cumbersome analytic techniques. Following oral intake, the blood cobalt concentration-time curve displays an absorptive half-life of 0.9 hrs, an elimination phase half-life of 3.9 hrs and a terminal elimination half-life of 22.9 hrs [15].

## Implications of the hypothesis

Antidoping laws exist to provide a safe and fair environment for participation in sport. These laws should prevent and protect athletes from subjecting themselves to health risks through the use of unsafe, but performance-enhancing compounds. An area of major controversy is the "sports supplement" industry, which is poorly regulated when compared with prescription drugs, but yet is a potential source of doping violations. In this regard, pharmacokinetic characteristics, easy availability through the chemical industry and low costs would make cobalt administration the ideal surrogate or complement for rHuEpo administration, turning out to be the most suited blood doping technique for athletes seeking to improve aerobic performances with little chance of testing positive. At variance with blood doping, cobalt is not mentioned in the WADA prohibited list [14]. Nevertheless, the definition of blood doping currently includes the use of autologous, homologous or heterologous blood or red blood cell products of any origin, other than for medical treatment, and each means artificially enhancing the uptake, transport or delivery of oxygen, including but not limited to perfluorochemicals, efaproxiral (RSR13) and modified hemoglobin products (hemoglobin-based blood substitutes, microencapsulated hemoglobin products). Thus, cobalt may be quantified through the use of bioassays that are comprised of either in vivo and/or in vitro measurements, though in vitro analyses are routinely performed in situations where in vivo analyses can not be obtained or in support of an in vivo monitoring program. Available techniques for measuring whole blood, serum, plasma or urinary cobalt involve analytic approaches, such as electrothermal atomic absorption spectrometry, extractive spectrophotometric determination, differential pulse anodic stripping voltammetry, neutron activation analysis, inductively coupled plasma-atomic emission spectrometry and x-ray fluorescence and gas chromatography-mass spectrometry [16-18], most of which are currently not practical for antidoping laboratories. Then, the little information available so far on cobalt metabolism in athletes hampers the appropriate interpretation of population data and the analysis of potential doping cases. Additional testing strategies, relaying on the identification of indirect biological effects of cobalt chloride administration such as activation of vascular endothelial growth factor (VEGF) gene transcription [19] or enhanced synthesis of delta-aminolevulinate [20], may be reliable alternatives, but will necessary entail a long and demanding process of clinical and analytical validation. More research on cobalt metabolism in athletes is compelling, along with implementation of effective strategies to unmask this potentially deleterious doping practice.

## Competing interests

The author(s) declare that they have no competing interests.

## Authors' contributions

GL conceived of the study, and participated in its design and coordination and helped to draft the manuscript; MF participated in study design and helped to draft the manuscript; GCG coordinated the study design and helped to draft the manuscript. All authors read and approved the final manuscript.
